# The Impact of Touch Restrictions in Small-Sided Games on Soccer Players' Passing, Receiving, and Ball-Touch Temporal Intervals

**DOI:** 10.5114/jhk/196142

**Published:** 2025-05-29

**Authors:** Mathias Rosten, Tore Kristian Aune, Terje Dalen

**Affiliations:** 1 Department of Physical Education and Sport Science, Faculty of Education and Arts, Nord University, Norway.

**Keywords:** team sports, game tactics, specificity, overload, intensity, quantity

## Abstract

The present study aimed to investigate how players’ technical actions were influenced by different touch restrictions in small-sided games (SSGs). Specifically, it analysed differences in the temporal intervals separating players' first and second touches across varied field zones—defensive, centre, and offensive—in SSGs. The experimental design involved twelve male junior soccer players playing under three distinct 4v4 SSG conditions: 1) a maximum of two touches per player; 2) a maximum of three touches per player; and 3) unrestricted play with no touch restrictions. Ten games of 4v4 SSGs (4 players + goalkeeper per team) were held for each condition, resulting in a total of 30 games of 4v4 SSGs investigated across eight distinct test sessions. Players had the shortest intervals between their first and second touches in the offensive zone, followed by the centre and then the defensive zones. Furthermore, the imposition of touch restrictions in SSGs led to a heightened frequency of passes compared to SSGs characterised by unrestricted gameplay; by contrast, SSGs featuring free play facilitated a greater number of receptions compared to SSGs with constraints on the number of touches. In conclusion, the current study outlines distinct differences in temporal intervals between the first and second touches of the ball across distinct field zones, notably showcasing a significant reduction in the time elapsing between touches as players neared their opponents’ goal.

## Introduction

In the world of soccer, small-sided games (SSGs) are widely recognised as a cornerstone of training methodologies; they are valued for their ability to replicate the specific demands encountered during competitive matches (Aguiar et al., 2012; Clemente et al., 2012). Characterised by reduced numbers of players and field dimensions relative to conventional match settings, SSGs are a time-efficient means of enhancing skills across various aspects (Michailidis, 2013). They are thought to advance the development of players' technical and tactical insights while simultaneously strengthening their aerobic capacities (Helgerud et al., 2001; Rampinini et al., 2007).

Coaches take advantage of the flexibility of SSGs by tailoring the structure in order to target specific physical, technical, and tactical competencies, manipulating variables such as the number of players, field dimensions, and gameplay regulations, including restrictions on how many ball touches are allowed (Michailidis, 2013). Several studies have been conducted to examine such manipulations and how they affect various physiological, technical, and tactical requirements in soccer (de Dios-Álvarez et al., 2024; Hill-Haas et al., 2009, 2011; Jones and Drust, 2007; Skalski et al., 2024; Rabano-Muñoz et al., 2023). For example, a consensus has emerged regarding the relationship between a reduced number of players and increased shot frequency per play sequence (Almeida et al., 2013; Katis and Kellis, 2009), as well as the increase in technical actions with reduced field dimensions (Casamichana and Castellano, 2010). Moreover, constraints imposed on ball touches in SSGs seem to have effects on the technical dynamics of gameplay. Specifically, games permitting unrestricted ball touches show greater tackling frequencies compared to those with touch restrictions (Dellal et al., 2012b; Owen et al., 2014). Furthermore, a marginally elevated incidence of ball loss accompanies touch limitations, impacting the efficacy of successful passes (measured as a percentage): notably, games with a maximum of one touch exhibit significantly lower success rates (49.8%) compared to those allowing two touches (67.7% and 69%) and unrestricted play (73.3% and 73.5%) (Dellal et al., 2011a, 2012b).

Given these findings, SSGs featuring varying degrees of touch restrictions emerge as a recommended training modality, characterised by a tendency to expose players to scenarios which are representative of match environments, thereby enhancing both the overload and specificity in training. Previous studies have explored the implications of imposing limitations on players’ ball touches, specifically comparing scenarios wherein players are restricted to either one or two touches per possession with scenarios of unrestricted play (Dellal et al., 2012a; Casamichana et al., 2014). Notably, these studies found that the imposition of a one-touch maximum constraint might reduce certain technical maneuvers intrinsic to soccer, including receiving the ball and then passing it. Forced one-touch play contributes to SSGs becoming less like match conditions, thereby violating the principle of specificity in training methodologies.

Being able to perform technical actions at a high speed is of great importance in soccer; if players can effectively pass the ball quickly, the team will most likely dominate the match even if the pressure from the opposing team is intense. The ability to perform technical actions in the shortest possible time is considered one of the most important characteristics for a soccer player to be competitive (Wang, 2013). While prior research has elucidated the role of players’ positional placements in field dynamics—particularly regarding running distances and frequency of technical engagements (Dellal et al., 2010, 2012b)—a notable gap persists concerning the understanding of positional effects on the pace and intensity during ball possession.

Hence, the overall purpose of the present study was to investigate how players’ technical actions were influenced by different touch restrictions in 4v4 + goalkeeper SSGs. More specifically it was hypothesized that there would be 1) a gradual decrease in temporal intervals separating players' first and second touches of the ball when closer to the opponent’s goal (defensive, centre, and offensive zones), and 2) an increase in the number of passes with increased touch restrictions. Such an investigation has the potential to provide insights into the nuanced interplay between positional factors, game configurations, and technical performance in the context of SSGs.

## Methods

### Participants

The study included 12 male junior soccer players, with an average age of 17.9 years (± 0.8 years). In terms of physical characteristics, players had an average height of 180.2 cm (± 4.9 cm) and average body mass of 72.8 kg (± 8.9 kg). All the participants played together in the same junior team in the first division for junior players, at different positions on the field, and had several years of experience (9.7 ± 1.7 years) with organised soccer training. The experiment was conducted during the pre-season, and the study capitalised on the participants’ pre-existing familiarity with 4v4 SSG formats and the utilisation of varied touch restriction protocols. Adherence to the ethical guidelines delineated in the Helsinki Declaration was maintained throughout the study. All participants and their parents or legal guardians (with regard to minors) provided informed consent, affirming their voluntary engagement in the research study and acknowledging their right to withdraw from participation at any time. The study was approved by the Norwegian Social Science Data Services (approval code: 46443; approval date: 29 January 2016).

### Design and Procedures

The study was designed to investigate the influence of varying touch constraints in 4v4 SSGs on the qualitative and quantitative aspects of passing proficiency, ball reception efficacy, and temporal intervals between players’ first and second touches across distinct field zones. To this end, passes were evaluated in terms of their quantity, directional distribution, and success rates, and ball receptions were scrutinised in terms of their frequency and directional patterns. Moreover, the time between players’ first and second ball touches was analysed as a proxy for the speed of ball control, defined by seconds elapsed, and players’ positional allocations on the field. The field was divided into defensive, centre, and offensive zones to enable nuanced analysis of positional dynamics. The experimental design involved three distinct 4v4 SSG conditions: 1) imposition of a maximum of two touches per player; 2) imposition of a maximum of three touches per player; and 3) unrestricted play with no touch restrictions. Ten games of 4v4 SSGs (4 players + goalkeeper) were held and investigated for each condition, resulting in a total of 30 games of 4v4 SSGs, conducted across eight distinct test sessions.

The playing surface had consistent dimensions across all 4v4 SSGs—namely 20 by 30 m, a medium size conducive to such gameplay (Rampinini et al., 2007). Markers delineating defensive, centre, and offensive zones were established using cones, with each zone spanning 10 m in length; the defensive zone was the zone next to the player’s own goal, the zone in the middle was the centre zone, and the zone adjacent to the opponent’s goal was the offensive zone. To ensure uniformity in environmental conditions, all games took place indoors, thereby standardising the surface characteristics, lighting, ambient temperatures, and atmospheric conditions across test sessions. Each 4v4 SSG comprised four players and one goalkeeper per team, with the technical actions of the goalkeepers excluded from the analysis. Before the 4v4 SSGs began, participants underwent an approximately 20-min warm-up session involving ball-handling drills. Game duration was fixed at four minutes, divided by four-minute intermissions between successive games. Team composition varied in each test session and was randomised to avoid potential bias. The specific touch restriction rule applying to the 4v4 SSG condition was explained to participants prior to each match; however, no guidance was offered by the test manager or instructor during gameplay sequences.

To hinder potential confounding effects arising from session fatigue or order bias, the sequence of conditions was counter-balanced across test sessions, with all three test conditions being administered in each session. This approach served to neutralise any undue influence stemming from individual session performance discrepancies and ensured equitable distribution of conditions throughout each session. Video documentation of all games was recorded by two cameras: one overhead camera (Canon LEGRIA HF R36) positioned diagonally behind one goal at a height of 5.2 m above the field, and a handheld camera (Sony HDR-XR155E) tracking player and ball movements. Subsequent analysis of the recorded footage enabled detailed examination of selected technical actions.

### Analysis

The video analysis was performed employing Kinovea software (version 08.15); the video footage was repeatedly and meticulously examined against pre-established criteria pertaining to passing, receiving, and the temporal interval between initial and subsequent ball contacts. The definitional constructs employed by Owen et al. (2004) were referenced for the categorising of passing and receiving actions. Specifically, passing was identified as the intentional transmission of the ball from one player to a teammate by any of a variety of techniques involving the foot, the thigh or the chest, encompassing diverse modes such as ground passes, high passes, chips, flicks, volleys, and variable distances; receiving was defined as the player achieving control and maintaining possession of the ball (Owen et al., 2004). Situations in which players failed to carry out other touches of the ball were not defined as receiving the ball.

To investigate differences in passing and receiving directions, this study used a set-up based on an analysis system drawn up by Thomas et al. (2009). This was used to distinguish among three different types of passing and receiving: forward, diagonal, and backward (Thomas et al., 2009).

Time between the first touch and the second touch of the ball was measured in seconds with precision of 0.04 s. The Kinovea functionality facilitated a frame-by-frame examination, enabling precise determination of when players touched the ball. Both the average duration throughout a gameplay sequence and the minimum time—signifying the shortest temporal interval between the first and second touches in a sequence—were subjected to analysis. The utilisation of defensive, centre, and offensive zones enabled an exploration of temporal disparities based on players’ field positions at the point of receiving the ball.

### Statistical Analysis

Given n = 12, α = 0.05, and 1 - β = 0.80, the minimum detectable effect size was *d* = 0.54, determined through a sensitivity power analysis for within-only repeated measures ANOVA using G*Power 3.1.9.7 (Faul et al., 2007). Initially, assessment of normality was conducted using the Shapiro-Wilk test. Following the normality test, a one-way repeated measures ANOVA accompanied by Bonferroni corrections was employed to scrutinise the within-subject effects across the 4v4 SSG conditions. For cases in which the sphericity assumption was violated, the Greenhouse-Geisser adjustments to the *p*-values were reported in the results. Subsequently, a two-way repeated measures ANOVA with Bonferroni correction was performed to investigate the interaction of the time between the first and second touches and the position zone on the field. For each model, effect size (Cohen’s *d*) was calculated as the difference in estimated marginal means between two conditions, divided by the SD derived from the standard error (SE ∙ √n). Effect sizes were interpreted according to Cohen (1988) as trivial <0.2, small ≥0.2, moderate ≥0.5, and large ≥0.8. Data are presented as mean ± standard deviation (SD). For all tests, the level for significance was set at *p* < 0.05. Statistical analysis was performed using SPSS 29.0.2.0 for Windows (SPSS Inc., Chicago, IL, USA).

## Results

The mean time interval between the first and second touches of the ball was shorter when closer to the opponent’s goal, as shown in Figure 1. Additionally, with respect to the players’ positional allocations at the point of ball reception, the shortest duration of time between two touches was consistently observed in the offensive zone, with the second-shortest duration seen in the centre zone and the longest duration seen in the defensive zone, as illustrated in Figure 1. The need for high speed of ball control (short time interval between the first and second touches) in zones near the opponent’s goal remained consistent across all three SSG conditions, as depicted in Figure 1.

The means for each 4-min play reveal a higher number of passes in SSGs with the maximum of two touches compared to 4v4 SSGs with free play, whereas SSGs with free play demonstrated a higher number of total receptions and receptions forward compared to 4v4 SSGs with the maximum of two touches (Table 1). Also, in SSGs with the maximum of two touches, players made significantly more passes during their first touch of the ball than in SSGs with free play (*p* < 0.01) and SSGs with the maximum of three touches (*p* < 0.05). There were no differences in the success rates of passes between SSGs with the maximum of two touches, SSGs with the maximum of three touches, and SSGs with free play (75.42%, 75.46%, and 74.61%, respectively). There were also significantly more passes during the first touch of the ball in SSGs with the maximum of three touches than in SSGs with free play (*p* < 0.01). The mean number of touches per ball possession per player was also significantly lower in SSGs with the maximum of two touches compared to SSGs with the maximum of three touches and SSGs with free play (*p* < 0.05) (1.54 ± 0.07 vs. 1.74 ± 0.13 and 2.39 ± 0.47, respectively).

**Table 1:** Differences in mean numbers and directions of passes and receptions, as well as time between 1^st^ and 2^nd^ touches, across the different 4v4 SSG conditions (mean ± SD, n = 12).

	4v4 SSGs with max. two touches	4v4 SSGs with max. three touches	4v4 SSGs with free play
Passing total (counts)	66.59 ± 11.99^*^	62.4 ± 1.59	53.8 ± 8.24
Passing backward (counts)	21.08 ± 6.28^*^	19.67 ± 4.76	15.17 ± 6.12
Passing diagonally (counts)	14.16 ± 4.71	13.37 ± 5.98	11.83 ± 3.43
Passing forward (counts)	30.58 ± 9.31	29.38 ± 10.50	27.00 ± 7.02
Receiving total (counts)	38.92 ± 8.32	43.25 ± 11.35	45.1 ± 11.10^§^
Receiving backward (counts)	5.67 ± 2.64	8.31± 4.03	7.07 ± 3.66
Receiving diagonally (counts)	12.75 ± 4.05	13.14 ± 4.39	11.94 ± 6.61
Receiving forward (counts)	20.50 ± 5.95	21.80 ± 6.96	24.68 ± 6.08^§^
Mean time between 1^st^ and 2^nd^ touch (s)	0.95 ± 0.06	0.92 ± 0.09	0.93 ± 0.07
Min. time between 1^st^ and 2^nd^ touch (s)	0.36 ± 0.08	0.35 ± 0.07	0.35 ± 0.07

* More than 4v4 SSG with free play (p < 0.05); § More than 4v4 SSG with the maximum of two touches (p < 0.05)

**Table 2:** Mean differences and effect sizes in the number and direction of passes and receptions, as well as the time between the first and second touches, were analyzed across different 4v4 small-sided game (SSG) conditions. Additionally, mean differences and effect sizes in the time between the first and second touches were examined in various zones on the pitch and under different SSG conditions (n = 12).

4v4 SSGs with…	max. two touches vs max three touches (Diff. EM–Cohen’s *d*)	max. two touches vs free play (Diff. EM–Cohen’s *d*)	max. three touches vs free play (Diff. EM–Cohen’s *d*)
Passing total	(4.17–0.35)	(12.75–1.07)	(8.58–0.52)
Passing backward	(2.17–0.26)	(6.67–0.83)	(4.50–0.64)
Passing diagonally	(0.80–0.15)	(2.33–0.41)	(1.54–0.24)
Passing forward	(1.20–0.21)	(3.58–0.57)	(2.38–0.33)
Receiving total	(4.33–0.61)	(6.17–0.82)	(1.83–0.19)
Receiving backward	(2.64–0.64)	(1.41–0.38)	(1.23–0.25)
Receiving diagonally	(0.39–0.09)	(0.81–0.11)	(1.21–0.27)
Receiving forward	(1.30–0.23)	(4.18–0.83)	(2.88–0.55)
Mean time (s) between 1^st^ and 2^nd^ touch	(0.024–0.26)	(0.015–0.19)	(0.009–0.09)
Min. time (s) between 1^st^ and 2^nd^ touch	(0.010–0.10)	(0.007–0.07)	(0.003–0.03)
Min. time between 1^st^ and 2^nd^ touch	Zone 1 vs. Zone 2 (Diff. EM–Cohen’s *d*)	Zone 1 vs. Zone 3 (Diff. EM–Cohen’s *d*)	Zone 2 vs. Zone 3 (Diff. EM–Cohen’s *d*)
SSGs with free play	(0.11–1.02)	(0.25–1.71)	(0.14–0.87)
SSGs with max. two touches	(0.11–0.87)	(0.28–2.94)	(0.17–1.56)
SSGs with max. three touches	(0.13–0.99)	(0.28–1.87)	(0.15–1.69)

## Discussion

The overall purpose of the present study was to investigate how players’ technical actions were influenced by different touch restrictions in a 4v4 + goalkeeper SSG. As hypothesized, the main finding was the gradual need for temporal efficiency in ball control, with players leaving the shortest time intervals between their first and second touches in the offensive zone, then in the centre zone, and the longest intervals in the defensive zone. Secondly, as hypothesized, the imposition of touch restrictions of two or three touches led to an increased frequency of passes compared to SSGs with unrestricted gameplay; by contrast, SSGs with free play demonstrated a greater number of receptions compared to SSGs with constraints on the number of touches.

The study findings outline significant differences across distinct field zones in the temporal intervals between the first and second touches of the ball, notably revealing a significant reduction in the time between touches closer to the opponent’s goal. Importantly, this observed pattern persisted across all three SSG conditions. In light of the principle of the overload (Bompa and Haff, 2009), these findings are significant, as players who frequently engage in ball receptions closer to the opponent’s goal during training are afforded more opportunities to improve their ability to navigate high-pressure scenarios within constrained timeframes, thereby fostering greater efficacy in real match situations through systematic exposure to stressors (Siff and Verkhoshansky, 1999). In this context, for a player accustomed to ample time between ball touches, a higher frequency of receptions in the offensive zone translates into greater demands, triggering a gradual progressive overload. This aligns with the assertion of Bompa and Haff (2009) concerning training loads, which posits that progress in adaptation requires a commensurate gradual, progressive, and escalating workload.

The diverse temporal dynamics between the first and second touches observed across the three zones can be ascribed to varying degrees of oppositional pressure. In a study on matches in the French top division, Dellal et al. (2010) found that attacking players had a greater proportion of ball losses and tackling losses compared to other positions on the field. Those authors explained it as due to the attacking players’ location in a more densely populated zone, in which they were often outnumbered by defensive players (Dellal et al., 2010). Even though their study investigated 11-a-side games, it could be argued that it is likely that something similar takes place in 4-a-side games, since the importance of defending one’s own goal is equal in both formats of the game. In addition, attacking players often play with their backs to their opponent’s goal, so they have a defensive player behind them when receiving the ball (Dellal et al., 2010). Analogously, this also occurs in 4v4 SSGs, as the interplay between players in the centre and offensive zones often originates from the goalkeeper or the deepest defender. Such interplay will often result in meeting an oncoming player, since the reduced field length results in minimal space behind the opponent’s defense. Consequently, players receiving the ball in densely populated zones, with an opposing player in close proximity, naturally face reduced timeframes to execute subsequent actions. The findings of this study reveal a lack of significant difference across the SSG conditions concerning both mean and minimum temporal intervals between the first and second ball touches. Such uniformity indicates that players at this level use the necessary temporal duration, irrespective of touch restrictions. Consequently, given these outcomes of the current investigation, it appears difficult to modulate the temporal dynamics between the first and second touches of the ball by implementing touch regulations dictating the permissible number of touches.

The study's outcomes also reveal a notable increase in the frequency of passes when restrictions on the permitted number of touches are imposed. One plausible rationale for this lies in the imperatives imposed by touch regulations, which compel players to rely more heavily on passing as a means of ball progression, as opposed to running or dribbling (Dellal et al., 2011b). This interpretation finds support in the increase in passes executed on the first touch of the ball in the context of 4v4 SSGs subjected to touch regulations, as observed in this study. Furthermore, the imposition of two- and three-touch constraints yielded a significantly greater number of ball possessions per player compared to the free play condition. These findings concur with those reported by Dellal and colleagues, who similarly concluded that touch restrictions—specifically those allowing one or two touches—resulted in more instances of ball possession per player compared to unrestricted gameplay (Dellal et al., 2011b, 2012b). The greater frequency of individual ball possessions within a given gameplay sequence increases the opportunities for players to pass the ball. SSGs serve as an effective platform for passing training due to their inherent specificity, in that they mirror various facets of real match situations. In contrast to conventional passing drills conducted in isolation, SSGs afford players the opportunity to practice their passing skills under pressure from opposing players, thereby enhancing the transferability of their acquired skills to identical match scenarios (Hill-Haas et al., 2008; Owen et al., 2014).

In the context of free play, players exhibited a significantly higher frequency of receptions compared to scenarios with the maximum of two touches, and a marginally greater frequency of receptions compared to scenarios with the maximum of three touches. In terms of refining receiving proficiency, free play emerges as a particularly effective setting, due to the heightened volume of repetitions of receiving it affords. This is particularly salient when contrasted against maximum-two touch restrictions, where a notably lower number of receptions is observed than under the free play condition. One plausible explanation for the higher incidence of receptions in free play lies in the tendency for passes to be more frequently executed on the first touch in SSGs featuring touch constraints, as evidenced by this study’s findings. Consequently, it can be inferred that the pace of passing between players is faster in games with two-touch restrictions compared to those with free play, wherein players tend to employ multiple touches before executing a pass. Notably, the study found a substantially lower average number of touches per instance of ball possession when touch restrictions were in place, specifically in scenarios allowing for two (1.54) and three (1.74) touches. This finding suggests that a significant proportion of passes are executed immediately upon receiving the ball. The number of touches per instance of ball possession holds considerable significance as a key determinant of technical proficiency in match situations, particularly in elite soccer contexts: Dellal et al. (2011b) found that elite players typically employed 1.74–2.24 touches per instance of ball possession during match play. Therefore, the observed reduction in touches per instance of ball possession in touch-restricted SSGs indicates a departure from typical elite player behavior, one which exhibits a more rapid and incisive passing approach as necessitated by touch constraints.

The present findings indicate that touch regulations of a maximum of two or three touches per player do not discernibly influence the success rate of passes. It can be assumed that no changes were seen in the success rate of passes because the pressure from the opponents does not vary notably among the different conditions. This contention finds support in the similarity of pass success rates across all three conditions, which fall within the range observed in match situations involving elite players in the French top division (68–78%) (Dellal et al., 2010).

The predominance of forward passes observed across all conditions can be attributed to the strategic imperative of advancing towards the opponent’s goal in order to create scoring opportunities. By contrast, there is a notable increase in backward passes in games featuring touch restrictions compared to games with free play. This phenomenon may stem from players lacking the option to turn with the ball in touch-regulated scenarios, compelling them to resort to backward passes as a means of retaining possession. Conversely, in free play, players possess the freedom to pivot with the ball, potentially removing the need for backward passes. In addition, the higher incidence of backward receptions under conditions featuring a maximum three-touch regulation compared to a maximal two-touch regulation suggests a propensity for players to prioritise ball retention over forward progression under the former condition. However, it is worth noting that in scenarios with touch restrictions, players may opt to execute passes on the first touch to avoid the risk of losing possession, potentially leading to instances without recorded receptions; this could contribute to the observed differences in the direction of receptions between conditions featuring different touch restrictions.

## Limitations of the Study

Some limitations accompany the present study. First, the investigation included only a single team with a relatively small sample size, and specific aspects of the team's training history—such as the type of coaching and the tactical philosophy of the team or the club—may have influenced the results. Additionally, the performance level of the team, being at the junior level, might also impact the findings. Future research would benefit from including players from both higher and lower performance levels to gain a more comprehensive understanding. Another limitation pertains to the characteristics of the small-sided games (SSGs) employed in the study. Only one type of SSG was analyzed, and future studies should explore how variations in SSG characteristics (e.g., the number of players, the playing area, etc.) influence players' technical actions. Furthermore, it is essential to acknowledge the potential limitations related to the quality and accuracy of the video analytics employed in the study. Future research could enhance validity by utilizing multiple cameras with higher sampling frequencies, particularly to more accurately capture the fastest movements.

## Practical Implications

For soccer coaches seeking to cultivate match-like passing scenarios characterised by the pace and oppositional pressure, this study underscores the efficacy of using restrictions on the permitted number of touches, which produce a higher frequency of passes per player per minute compared to free play scenarios. The use of SSGs for reception training confers a high degree of specificity, as players contend with dynamic pressure from opponents, which necessitates tactical considerations of teammates’ and opponents’ positions vis-à-vis their own along with stringent technical requisites for executing receptions effectively.

If the training objective is to prioritise maximising the number of ball receptions during gameplay sequences, then free play is the condition best suited to this aim. Notably, SSG manipulations involving touch restrictions do not demonstrably influence the temporal intervals between the first and second ball touches; instead, the player’s positional placement on the field upon receiving the ball exerts a discernible impact on this temporal dynamic. Consequently, rotating players’ positions in 4v4 SSGs has potential as a strategic imperative, as it encourages players to challenge themselves to minimise the duration between touches, thereby fostering a multifaceted skill-set adaptable to various positional exigencies on the field.

## Conclusions

The integration of regulations restricting the permitted number of touches in 4v4 SSGs offers a viable avenue for increasing the frequency of passing repetitions without compromising success rates. For their part, free play conditions afford the highest incidence of receptions in 4v4 SSGs, thereby increasing the number of repetitions in each gameplay sequence. Touch regulations demonstrate no discernible significance regarding the temporal intervals between the first and second ball touches; however, the positional orientation of players upon receiving the ball is noteworthy as an important factor, with the offensive zone hosting the shortest duration between these touches. These findings of the current study underscore the nuanced interplay between touch regulations, positional dynamics, and temporal aspects of gameplay in the context of 4v4 SSGs, and they provide valuable insights for coaches and practitioners seeking to optimise training methodologies that improve passing proficiency in soccer.
